# The Failure of *R*_0_

**DOI:** 10.1155/2011/527610

**Published:** 2011-08-16

**Authors:** Jing Li, Daniel Blakeley, Robert J. Smith?

**Affiliations:** ^1^Department of Mathematics, Pennsylvania State University, University Park, State College, PA 16802, USA; ^2^School of Mathematics and Statistics, University of Sheffield, Hicks Building, Hounsfield Road, Sheffield S3 7RH, UK; ^3^Department of Mathematics and Faculty of Medicine, The University of Ottawa, 585 King Edward Avenue, Ottawa ON, Canada K1N 6N5

## Abstract

The basic reproductive ratio, *R*_0_, is one of the fundamental concepts in mathematical biology. It is a threshold parameter, intended to quantify the spread of disease by estimating the average number of secondary infections in a wholly susceptible population, giving an indication of the invasion strength of an epidemic: if *R*_0_ < 1, the disease dies out, whereas if *R*_0_ > 1, the disease persists. *R*_0_ has been widely used as a measure of disease strength to estimate the effectiveness of control measures and to form the backbone of disease-management policy. However, in almost every aspect that matters, *R*_0_ is flawed. Diseases can persist with *R*_0_ < 1, while diseases with *R*_0_ > 1 can die out. We show that the same model of malaria gives many different values of *R*_0_, depending on the method used, with the sole common property that they have a threshold at 1. We also survey estimated values of *R*_0_ for a variety of diseases, and examine some of the alternatives that have been proposed. If *R*_0_ is to be used, it must be accompanied by caveats about the method of calculation, underlying model assumptions and evidence that it is actually a threshold. Otherwise, the concept is meaningless.

## 1. Introduction

The basic reproductive ratio—also known as the basic reproductive number, the basic reproduction number, the control reproduction number, or *R*_0_—is one of the foremost concepts in epidemiology [1–3]. *R*_0_ is the most widely used epidemiological measurement of the transmission potential in a given population [4]. It is a measure of initial disease spread, such that if *R*_0_ > 1, then the disease can invade an otherwise susceptible population and hence persist, whereas if *R*_0_ < 1, the disease cannot successfully invade and will die out. The concept is defined as the number of secondary infections produced by a single infectious individual in an otherwise susceptible population [5].

Despite its place at the forefront of mathematical epidemiology, the concept of *R*_0_ is deeply flawed. Defining *R*_0_ proves to be significantly more difficult than it appears. Few epidemics are ever observed at the moment an infected individual enters a susceptible population, so calculating the value of *R*_0_ for a specific disease relies on secondary methods. There are many methods to calculate *R*_0_ from mathematical models, few of which agree with each other and few of which produce the average number of secondary infections. Methods to calculate *R*_0_ from theoretical models include the survival function, the next-generation method, the eigenvalues of the Jacobian matrix, the existence of the endemic equilibrium, and the constant term of the characteristic polynomial. *R*_0_ can also be estimated from epidemiological data via the number of susceptibles at endemic equilibrium, the average age at infection, the final size equation and calculation from the intrinsic growth rate. For an overview, see Heffernan et al. [2].

Furthermore, there are many diseases that can persist with *R*_0_ < 1, while diseases with *R*_0_ > 1 can die out, reducing the utility of the concept as a threshold. *R*_0_ is also used as a measure of eradication for a disease that is endemic, but issues such as backward bifurcations, stochastic effects, and networks of spatial spread mean that an invasion threshold does not necessarily coincide with a persistence threshold. This results in a reduction of the usefulness of *R*_0_. For example, it is possible that a disease can persist in a population when already present but would not be strong enough to invade. Finally, the threshold value that is usually calculated is rarely the average number of secondary infections, diluting the usefulness of this concept even further. 

In this paper, we outline the problems with *R*_0_ and examine a number of alternatives that have been proposed. We include a worked example of malaria to demonstrate the many different results that the various methods give for the same model. Finally, we survey some of the recent uses of *R*_0_ in the literature. The number of articles that use *R*_0_ likely numbers in the tens of thousands, so an exhaustive review is not feasible. We have restricted ourselves to articles published since 2005 and which include interesting or novel explorations of *R*_0_.

## 2. Methods for Calculating *R*_0_

In this section, we identify some of the more popular methods (although by no means all) used to calculate *R*_0_. We also describe the limitations that each method presents and demonstrate one of the core problems with *R*_0_. Specifically, we address a key problem with *R*_0_: how do biologists make sense of it from mathematical models? (See, for example, the puzzled discussion in van den Bosch et al. [6].) 

 Although the “*R*” in *R*_0_ is derived from “reproductive”, based on the original formulation of the concept as the average number of secondary infections, many thresholds have been denoted by “*R*_0_”, even when they are not related to the average number of secondary infections. Thus, in keeping with the notation, we will use the notation *R*_0,*X*_ to denote an *R*_0_-like surrogate associated with a particular method, symbolised by *X*.

### 2.1. The Survival Function

The survival function is given by

(1) $$\begin{array}{cc} R_{0,S} & =\int_{0}^{\infty }\left(\begin{array}{c} \text{the average number of susceptible}\\ \text{individuals that an infected individual}\\ \text{newly infects per unit time}\\ \text{when infected for total time }a \end{array}\right)\\ & \;\;\times \left(\begin{array}{c} \text{the probability that a}\\ \text{newly infected individual}\\ \text{remains infectious}\\ \text{for at least time }a \end{array}\right)da \end{array}$$

**Figure 1:**
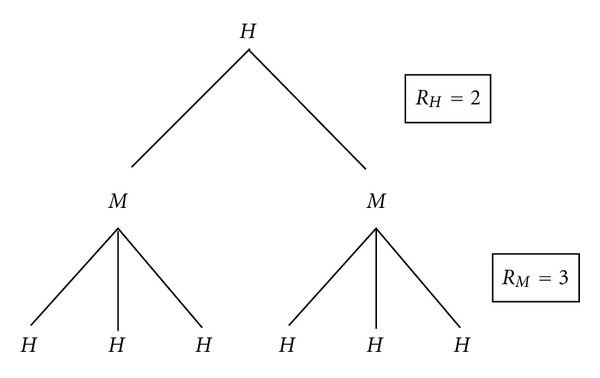
A example of a two-stage basic reproductive ratio. A single human infects *R*_*H*_ = 2 mosquitoes, each of whom subsequently infects *R*_*M*_ = 3 humans. Thus, a single human results in *R*_0_ = 6 infected humans. The next-generation method instead estimates $R_{0,N}=\sqrt{6}$ infected individuals in the subsequent generation, regardless of whether that generation is human or mosquito; *R*_0,*N*_ is the geometric mean of *R*_*H*_ = 2 and *R*_*M*_ = 3. (Reproduced with permission from Smith? [7]).

The survival function has the advantage that it always produces the average number of secondary individuals infected by a single infected individual, in the same class. Thus, in Figure 1, where one human infects two mosquitoes, who each infect three humans, the survival function produces *R*_0_ = 6. This is the number of humans infected by a single infected human via mosquitoes; or, equivalently, the number of mosquitoes infected by a single mosquito via humans. The survival function is a generalised method of calculating the basic reproductive ratio that is not restricted to ODEs.

However, determining the individual probabilities can be cumbersome, especially if multiple states are involved. For a vector-borne infection such as malaria, with two infection states (human and mosquito), calculating the first probability involves determining the probability that a human infected at time 0 exists at time *t*, the probability that a human infected for total time *t* infects a mosquito and the probability that an infected mosquito lives to be age *a* − *t*, where 0 ≤ *t* ≤ *a* [2]. For diseases with more states, such as Guinea Worm disease, where there is a waterborne parasite, which can attach itself to copepods, which in turn are ingested by humans and which subsequently grow into an internal nematode, the calculations of the survival probabilities become unwieldy.

Thus, although this method always produces the correct *R*_0_, in practice, it is difficult to use. This is especially true for models with sufficient complexity, which are often those encountered most frequently.

### 2.2. The Jacobian

The Jacobian matrix is used to linearise a nonlinear system of differential equations. Around the disease-free equilibrium, the linear system will have the same stability properties as the nonlinear system if it is hyperbolic; that is, if no eigenvalues have zero real part. In particular, if all eigenvalues have negative real part, then the equilibrium is stable, whereas if there is an eigenvalue with positive real part, the equilibrium is unstable.

It follows that a threshold is *λ*_max_ = 0, where *λ*_max_ is the largest eigenvalue of the Jacobian matrix (or the largest real part if the eigenvalues are complex). However, for a system of *n* differential equations, this requires solving an *n*th order polynomial, which may be impossible. Furthermore, rearranging the condition *λ*_max_ = 0 to produce a threshold *R*_0,*J*_ = 1 is not a unique process and does not always produce the average number of secondary infections. 

### 2.3. Constant Term of the Characteristic Polynomial

When *λ*_max_ = 0, the constant term of the characteristic polynomial will be zero. However, the reverse is not true, as the polynomial could have both zero and positive roots. If the characteristic polynomial is

(2) $$\begin{array}{cc} \lambda^{n}+a_{n-1}\lambda^{n-1}+\cdots +a_{1}\lambda +a_{0} & =0 \end{array},$$

then *a*_0_ = 0 is a threshold if *a*_*j*_ ≥ 0 for all *j*. More generally, the Routh-Hurwitz condition allows the coefficients to take on other signs, under certain restrictions, but the nonconstant coefficients all being positive is a sufficient condition. Another sufficient condition is that *a*_*j*_ ≥ 0 under the constraint *a*_0_ = 0 (so that the largest eigenvalue at *a*_0_ = 0 is 0).

This method is significantly easier to use than finding the largest eigenvalue, although verifying that *a*_0_ = 0 necessarily corresponds to the largest eigenvalue can become complicated for some models. However, similar to the above, rearranging *a*_0_ = 0 to produce *R*_0,*C*_ = 1 is not a unique process and does not always produce the average number of secondary infections.

### 2.4. The Next-Generation Method

The next-generation method, developed by Diekmann et al. [8] and Diekmann and Heesterbeek [9], and popularised by van den Driessche and Watmough [10], is a generalisation of the Jacobian method. It is significantly easier to use than Jacobian-based methods, since it only requires the infection states (such as the exposed class, the infected class and the asymptomatically infected class) and ignores all other states (such as susceptible and recovered individuals). This keeps the size of the matrices relatively manageable.

However, the next-generation method suffers from a lack of uniqueness. In order to determine the matrices *F* and *V* (where *F* accounts for the “new” infections and *V* accounts for the transfer between infected compartments), biological insight must be used in order to decide which terms count as “new” infections and which terms are transfer terms. While this may seem intuitive for most models, it is easy to construct a counterexample

(3) $$\begin{array}{cc} I^{\prime } & =\beta SI-\mu I\\ & =\beta SI+5I-5I-\mu I. \end{array}$$

Here, the term +5*I* might represent, for example, new infections arising from vertical transmission, whereas the term −5*I* might represent a disease-specific death rate. Although this construction is clearly arbitrary, it demonstrates that identifying “new” infections is not a unique process and relies on the modeller's judgement.

We would then have

(4) $$\begin{array}{cc} F & =\beta S+5,\;\;V=5+\mu , \end{array}$$

and thus

(5) $$\begin{array}{cc} R_{0,5} & =\frac{\beta S+5}{5+\mu }.\; \end{array}$$

This has the same threshold property as *R*_0,*N*_ = *βS*/*μ*, but is clearly not the average number of secondary infections. In essence, the next-generation method is a mathematical generalisation of putting the negative values on one side and dividing (this is what *V* ^−1^ is) so that the eigenvalue threshold at zero is transformed into an *R*_0_-like threshold at one. However, as we have seen, this does not produce a unique result.

van den Driessche and Watmough [10] note that other decompositions of *F* and *V* can be chosen, which lead to different values for *R*_0,*N*_. They claim that only one choice of *F* is epidemiologically correct. However, this is not true, as the above example shows. Furthermore, it means that the definition of *R*_0_ relies upon the judgement of the modeller as to what “epidemiologically correct” means.

Furthermore, the next-generation method does not produce the number of humans infected by a single human if there is an intermediate host, but rather the geometric mean of the number of infections per generation.

For example, consider a mosquito-borne disease where humans infect two mosquitoes, while mosquitoes infect three humans, as shown in Figure 1. For convenience, label these *R*_*H*_ = 2 and *R*_*M*_ = 3. Then the number of humans infected from a primary human (via mosquitoes) is *R*_0_ = 2 × 3 = 6. (This is also the value calculated by the survival function.)

However, the next-generation method would calculate $R_{0,N}=\sqrt{6}$, which is a weighted average ($2<\sqrt{6}<3$) of the number of infectives each individual produces in the next infection event. While mathematically sound, it is questionable whether this is biologically meaningful.

This example could be extended. Consider a three-stage disease, such as tularemia, where ticks may transmit between humans and livestock, but humans may also be infected by eating livestock. Suppose that a single human directly infects two ticks (so *R*_*H*_ = 2), each tick infects four animals (so *R*_*T*_ = 4) and each animal infects three humans (so *R*_*A*_ = 3). Then, a single human has resulted in

(6) $$\begin{array}{c} R_{0}=2\times 4\times 3=24 \end{array}$$

infected humans. However, $R_{0,N}=\sqrt[{3}]{24}\approx 2.88$, since there are three infection stages. As the number of infection stages increase, *R*_0,*N*_ becomes a progressively higher surd.

 The next-generation method is likely the most frequently used method to calculate *R*_0_. It has been used extensively to calculate *R*_0_-like values from host-vector models (see Wonham et al. [11], Gaff et al. [12] or Gubbins et al. [13]). However, it does not produce the number of newly infected individuals in the same infection class and does not always produce the average number of secondary infections.

### 2.5. The Graph-Theoretic Method

In de Camino-Beck et al. [14], a graph-theoretic method for calculating *R*_0_ is given. Starting from the definition of *R*_0_ = *ρ*(*FV* ^−1^), they derived a series of rules for reducing the digraph associated with *Fλ* ^−1^ − *V* to a digraph with zero weight, from which *λ* = *R*_0_. The rules are as follows.

**Rule 1**

To reduce the loop −*a*_*ii*_ < 0 to −1 at node *i*, every arc entering *i* has weight divided by *a*_*ii*_.

**Rule 2**

For a trivial node *i* on a path *j* → *i* → *k*, the two arcs are replaced by *j* → *k* with weight equal to the product of weights on arc *j* → *i* and *i* → *k*. Weights on multiple arcs *j* → *k* are added.

The graph-theoretic method has the advantage that it avoids calculation of *V* ^−1^, which may be complicated for large systems. However, it always produces the same threshold value as the next-generation method.

They considered the simple vector-host model

(7) $$\begin{array}{cc} \frac{dI}{dt} & =\beta_{s}SW-\left(b+\gamma \right)I,\\ \frac{dW}{dt} & =\beta_{m}MI-cW,\\ \frac{dS}{dt} & =b-bS+\gamma I-\beta_{s}SW,\\ \frac{dM}{dt} & =c-cM-\beta_{m}MI, \end{array}$$

where *I*, *W*, *S*, and *M* are proportions of infective hosts, infective vectors, susceptible hosts, and susceptible vectors, respectively, *b* is the host birth and death rate, *γ* is the host recovery rate, *c* is the vector birth and death rate, and *β*_*s*_ and *β*_*m*_ are disease transmission coefficients. The disease-free equilibrium is (0,0, 1,1)^*T*^. They found matrices

(8) $$\begin{array}{cc} F & =\left(\begin{array}{cc} 0 & \beta_{s}\\ \beta_{m} & 0 \end{array}\right),\;\;V=\left(\begin{array}{cc} b+\gamma & 0\\ 0 & c \end{array}\right). \end{array}$$

From this, they produced a digraph of *Fλ* ^−1^ − *V* and concluded that

(9) $$\begin{array}{cc} R_{0} & =\sqrt{\frac{\beta_{m}\beta_{s}}{c\left(b+\gamma \right)}}. \end{array}$$

However, this method still contains the same issues as the next-generation method. The *R*_0_ value calculated is not the number of humans infected by a single human, but rather the (less biologically meaningful) geometric mean of the number of humans infected by the vector and the number of vectors infected by a human. Furthermore, the creation of *F* and *V* is not a unique process, as we have seen above, and does not always produce the average number of secondary infections. 

For example, consider the mathematically equivalent model

(10) $$\begin{array}{cc} \frac{dI}{dt} & =\left(\beta_{s}+9\right)SW-9SW-\left(b+\gamma \right)I,\\ \frac{dW}{dt} & =\beta_{m}MI-cW,\\ \frac{dS}{dt} & =b-bS+\gamma I-\beta_{s}SW,\\ \frac{dM}{dt} & =c-cM-\beta_{m}MI, \end{array}$$

with matrices

(11) $$\begin{array}{cc} F & =\left(\begin{array}{cc} 0 & \beta_{s}+9\\ \beta_{m} & 0 \end{array}\right),\;\;V=\left(\begin{array}{cc} b+\gamma & 9\\ 0 & c \end{array}\right). \end{array}$$

**Figure 2:**
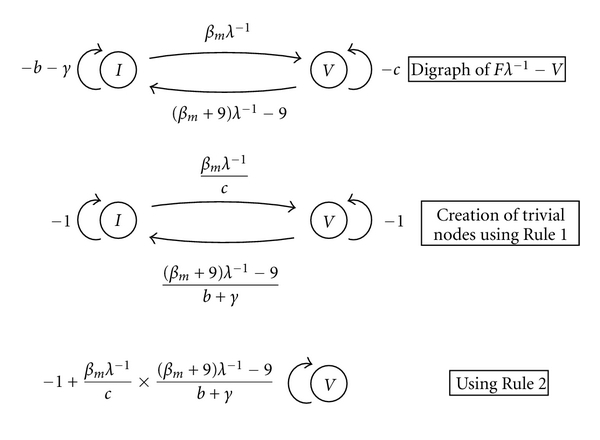
The graph-theoretical method of de Camino-Beck et al. [14] applied to a vector-host model may not produce a unique *R*_0_.

The graph reduction then proceeds as in Figure 2. It follows (either by the graph-reduction method or using the conventional next-generation method) that

(12) $$\begin{array}{cc} R_{0} & =\frac{1}{2}\left[\frac{9\left(\beta_{s}+9\right)}{c\left(b+\gamma \right)}+\sqrt{\frac{81{\left(\beta_{s}+9\right)}^{2}}{c^{2}{\left(b+\gamma \right)}^{2}}+\frac{4\beta_{m}\left(\beta_{s}+9\right)}{c\left(b+\gamma \right)}}\;\right]. \end{array}$$

Thus, the graph-theoretic method inherits the existing problems from the next-generation method.

### 2.6. Existence of the Endemic Equilibrium

*R*_0_ can also be calculated from the endemic equilibrium, in the case where there is a bifurcation at *R*_0_ = 1 such that the endemic equilibrium does not exist for *R*_0_ < 1. The existence of the endemic equilibrium is, thus, a threshold that can be rearranged to produce an *R*_0_-like surrogate.

However, for many models, calculating the endemic equilibrium can be quite difficult. Furthermore, in the case of a backward bifurcation, the endemic equilibrium still exists for *R*_0_ < 1 (in fact, two endemic equilibria exist in this case, one of which is stable and the other unstable). It follows that the endemic equilibrium is not a useful general method for calculating *R*_0_ and does not always produce the average number of secondary infections. 

 van den Bosch et al. [6] use the endemic equilibrium to determine a threshold given by $\hat{I}=(1/\alpha )(\mu q-\mu +\alpha K)$ so that $\hat{I}>0$ if *μq* − *μ* + *αK* > 0 (where $\hat{I}$ represents infected plants at the endemic equilibrium, *α* is the transmissibility, *μ* is the death rate, *q* is the fraction of infectious seeds, and *K* is the total plant population density). They show that two different rearrangements of this inequality give

(13) $$\begin{array}{c} \frac{\alpha }{\mu \left(1-q\right)}K>1\;\text{or}\;\frac{\alpha }{\mu }K+q>1, \end{array}$$

and note that either would suffice as an *R*_0_ value, but were unable to resolve the question of which was appropriate.

### 2.7. Summary of *R*_0_ Methods

In summary, there are many methods available for calculating *R*_0_, but few of them agree with each other and almost none reliably calculate the average number of secondary infections in a wholly susceptible population. The only method which does is the survival function, but this method is too cumbersome to be used except for the simplest of models.

## 3. A Worked Example

In this section, we take a sample model and apply the various methods for calculating *R*_0_ to it. We show that each method can produce a different result, all of which have the property that they have an outbreak threshold at *R*_0_ = 1 but otherwise bear little relation to one another.

Consider the following model for malaria:

(14) $$\begin{array}{cc} H_{S}^{\prime } & =\Lambda_{H}-\beta_{MH}M_{I}H_{S}-\mu_{H}H_{S},\\ H_{I}^{\prime } & =\beta_{MH}M_{I}H_{S}-\left(\mu_{H}+\sigma \right)H_{I},\\ M_{S}^{\prime } & =\Lambda_{M}-\beta_{HM}M_{S}H_{I}-\mu_{M}M_{S},\\ M_{I}^{\prime } & =\beta_{HM}M_{S}H_{I}-\mu_{M}M_{I}. \end{array}$$

**Figure 3:**
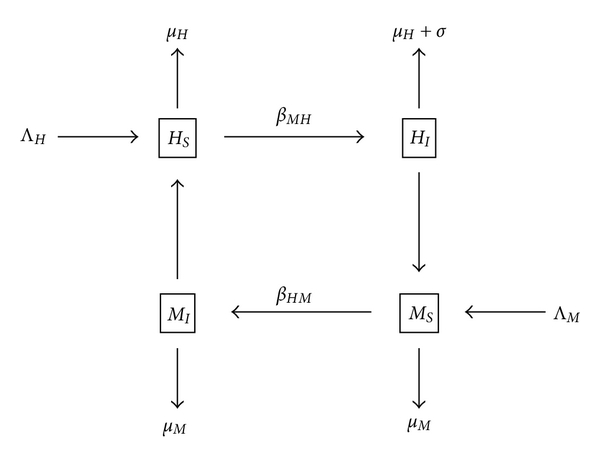
A standard model of malaria. Humans can be susceptible or infected, with birth rate Λ_*H*_, background death rate *μ*_*H*_ and disease-specific death rate *σ*. Mosquitoes can be susceptible or infected, with birth rate Λ_*M*_ and background death rate *μ*_*M*_.

Humans may be susceptible or infected (*H*_*S*_ and *H*_*I*_, resp.), while mosquitoes may be susceptible or infected (*M*_*S*_ and *M*_*I*_, resp.). The birth rates are Λ_*H*_ for humans and Λ_*M*_ for mosquitoes. The background death rates are *μ*_*H*_ and *μ*_*M*_ for humans and mosquitoes, respectively, while the disease-specific death rate for humans is *σ*. The transmission rate from mosquitoes to humans is *β*_*MH*_, while the transmission rate from humans to mosquitoes is *β*_*HM*_. See Figure 3.

The Jacobian matrix at the disease-free equilibrium is

(15) $$\begin{array}{cc} J & =\left[\begin{array}{cccc} -\mu_{H} & 0 & 0 & -\beta_{MH}{\bar{H}}_{S}\\ 0 & -\mu_{H}-\sigma & 0 & \beta_{MH}{\bar{H}}_{S}\\ 0 & -\beta_{HM}{\bar{M}}_{S} & -\mu_{M} & 0\\ 0 & \beta_{HM}{\bar{M}}_{S} & 0 & -\mu_{M} \end{array}\right], \end{array}$$

where ${\bar{H}}_{S}$ and ${\bar{M}}_{S}$ are the equilibrium values of susceptible humans and mosquitoes, respectively. Thus, the nontrivial part of the characteristic polynomial satisfies

(16) $$\begin{array}{c} \lambda^{2}+\left(\mu_{H}+\sigma +\mu_{M}\right)\lambda +\mu_{M}\left(\mu_{H}+\sigma \right)-\beta_{HM}\beta_{MH}{\bar{H}}_{S}{\bar{M}}_{S}=0. \end{array}$$

Using the Jacobian method, the largest eigenvalue is

(17) $$\begin{array}{cc} \lambda_{\max\;} & =\frac{1}{2}[-\mu_{H}-\sigma -\mu_{M}\;\\ & \;\;\;+\sqrt{{\left(\mu_{M}-\sigma -\mu_{H}\right)}^{2}+4\beta_{HM}\beta_{MH}{\bar{H}}_{S}{\bar{M}}_{S}}\;]. \end{array}$$

Rearranging *λ*_max_ = 0, we have

(18) $$\begin{array}{cc} R_{0,J} & =\sqrt{\frac{{\left(\mu_{M}-\sigma -\mu_{H}\right)}^{2}+4\beta_{HM}\beta_{MH}{\bar{H}}_{S}{\bar{M}}_{S}}{{\left(\mu_{M}+\sigma +\mu_{M}\right)}^{2}}}. \end{array}$$

Conversely, since the nonconstant coefficients of the characteristic polynomial are positive, all eigenvalues will be negative if the constant term of the characteristic polynomial is positive, whereas there will be an eigenvalue with positive real part if the constant term is negative. Rearranging, we have

(19) $$\begin{array}{cc} R_{0,C} & =\frac{\beta_{MH}\beta_{HM}{\bar{H}}_{S}{\bar{M}}_{S}}{\left(\mu_{H}+\sigma \right)\mu_{M}}. \end{array}$$

This is not the only rearrangement possible, so, like the nonuniqueness of the next-generation method, it is possible to rearrange by adding and subtracting arbitrary constants. Thus, for example,

(20) $$\begin{array}{cc} R_{0,9} & =\frac{\beta_{MH}\beta_{HM}{\bar{H}}_{S}{\bar{M}}_{S}+9}{\left(\mu_{H}+\sigma \right)\mu_{M}+9} \end{array}$$

is also a measure of disease spread with the property that the disease persists if *R*_0,9_ > 1 and is eradicated if *R*_0,9_ < 1.

Other rearrangements are possible, so long as the threshold at 0 is transformed into a threshold at 1. Thus,

(21) $$\begin{array}{cc} R_{0,e} & =\exp\;\left(\beta_{MH}\beta_{HM}{\bar{H}}_{S}{\bar{M}}_{S}-\left(\mu_{H}+\sigma \right)\mu_{M}\right) \end{array}$$

has the same threshold property. A generalised version of this formulation was used in Smith? et al. [15] called *T*_0_.

The endemic equilibrium satisfies

(22) $$\begin{array}{cc} {\hat{M}}_{I} & =\frac{\beta_{HM}{\hat{M}}_{S}{\hat{H}}_{I}}{\mu_{M}},\\ {\hat{M}}_{S} & =\frac{\Lambda_{M}}{\beta_{HM}{\hat{H}}_{I}+\mu_{M}},\\ {\hat{H}}_{S} & =\frac{\left(\mu_{M}+\sigma \right)\mu_{M}\left(\beta_{HM}{\hat{H}}_{I}+\mu_{M}\right)}{\beta_{MH}\beta_{HM}\Lambda_{M}},\\ {\hat{H}}_{I} & =\frac{\Lambda_{H}\Lambda_{M}\beta_{MH}\beta_{HM}-\mu_{H}\left(\mu_{H}+\sigma \right)\mu_{M}^{2}}{\Lambda_{M}\beta_{MH}\beta_{HM}\left(\mu_{H}+\sigma \right)+\beta_{HM}\mu_{H}\mu_{M}\left(\mu_{H}+\sigma \right)}. \end{array}$$

It follows that there will be a biologically meaningful endemic equilibrium if ${\hat{H}}_{I}>0$, or

(23) $$\begin{array}{cc} R_{0,E} & =\frac{\beta_{MH}\beta_{HM}{\bar{H}}_{S}{\bar{M}}_{S}}{\left(\mu_{H}+\sigma \right)\mu_{M}}>1, \end{array}$$

since ${\bar{M}}_{S}=\Lambda_{M}/\mu_{M}$ and ${\bar{H}}_{S}=\Lambda_{H}/\mu_{H}$. As above, this is not the only rearrangement possible.

The next-generation matrices are

(24) $$F=\left[\begin{array}{cc} 0 & \beta_{MH}{\bar{H}}_{S}\\ \beta_{HM}{\bar{M}}_{S} & 0 \end{array}\right],\;\;V=\left[\begin{array}{cc} \mu_{H}+\sigma & 0\\ 0 & \mu_{M} \end{array}\right],$$

since the next-generation method only considered the two infected classes. Then, using the next-generation method,

(25) $$\begin{array}{cc} R_{0,N} & =\sqrt{\frac{\beta_{MH}\beta_{HM}{\bar{H}}_{S}{\bar{M}}_{S}}{\left(\mu_{H}+\sigma \right)\mu_{M}}}. \end{array}$$

**Figure 4:**
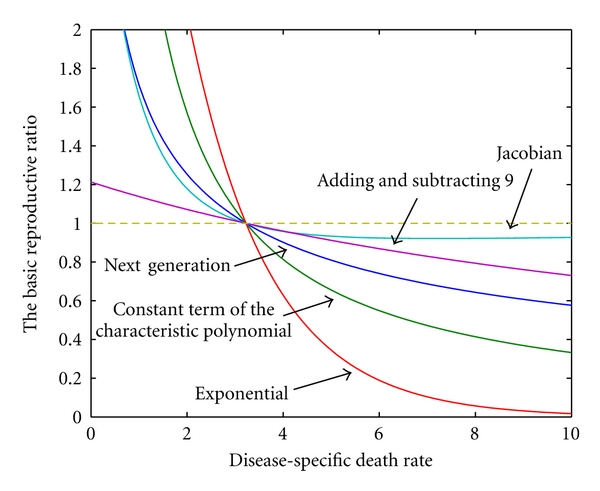
The changing face of *R*_0_. All expressions for *R*_0_ were generated from the same model, using standard techniques. As the death rate varies, all have the property that if *R*_0_ > 1 then an outbreak will occur, whereas if *R*_0_ < 1, then the disease will be eradicated. However, aside from the common threshold at *R*_0_ = 1, none of these expressions are equal to each other. The curve labelled “Jacobian” illustrates *R*_0,*J*_, as given by (18). The curve labelled “Adding and subtracting 9” illustrates the value *R*_0,9_ as given by (20). The curve labelled “Next generation” illustrates *R*_0,*N*_, as given by (25). The curve labelled “Constant term of the characteristic polynomial” illustrates *R*_0,*C*_, as given by (19). The curve labelled “Exponential” illustrates *R*_0,*e*_, as given by (21). In this case, the values are ${\bar{H}}_{S}=45$, ${\bar{M}}_{S}=30$, *β*_*MH*_ = 0.05, *β*_*HM*_ = 0.03, *μ*_*H*_ = 0.15 and *μ*_*M*_ = 0.6.

Thus, for the same model, *R*_0,*N*_, *R*_0,*J*_, *R*_0,*C*_, *R*_0,9_, and *R*_0,*e*_ are all distinct. Although *R*_0,*E*_ = *R*_0,*C*_ in this case, this does not hold in general. Note that *R*_0,*C*_ and *R*_0,*E*_ produce the number of infected humans per infected human (or, equivalently, the number of infected mosquitoes per infected mosquito), but this is also not necessarily true in general.  Figure 4 illustrates how these *R*_0_-like expressions vary with *σ*, when all other parameters are held constant.

 Anecdotally, we have found that most biologists believe that there is one and only one *R*_0_ value for a given disease and, in order to verify an *R*_0_ is correct, all that is required is to check that it has a threshold at 1. We have demonstrated here that *R*_0_ is not a unique concept. Indeed, it is straightforward to construct simple variations that satisfy the threshold criterion at 1. Furthermore, since multiple methods calculate multiple thresholds, it follows that the vast majority of them cannot be the average number of secondary infections. However, the nonuniqueness is not the only problem associated with the concept, as we will demonstrate.

## 4. Backward Bifurcations

Backward bifurcations occur when multiple stable equilibria coexist for *R*_0_ < 1. This presents a serious complication when a disease is already endemic, since lowering the basic reproduction number below 1 may no longer be a viable control measure; hence, different prevention and control measures may have to be considered. In particular, a backward bifurcation makes the system more complicated, since the behaviour now depends on the initial conditions.

**Figure 5:**
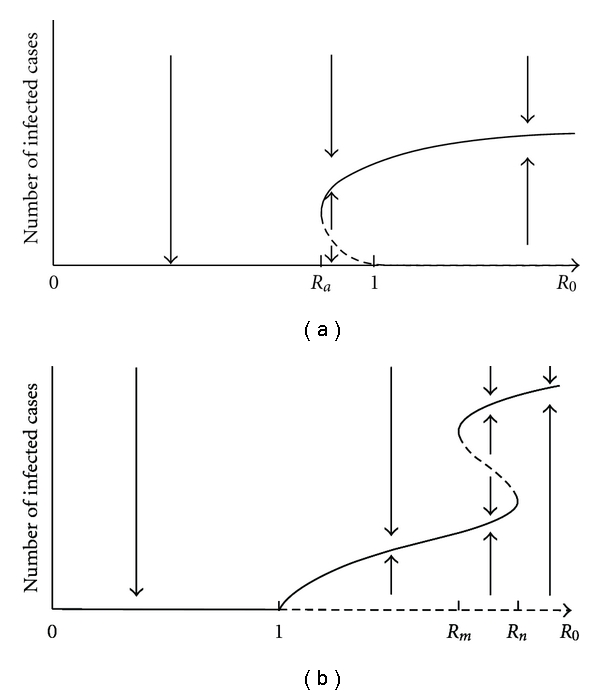
The effects of backward bifurcations. Solid curves indicate stable equilibria, while dashed curves indicate unstable equilibria. (a) A backward bifurcation at *R*_0_ = 1 may result in persistence of the disease when *R*_0_ < 1. There is a point *R*_*a*_ < 1 such that the endemic equilibrium exists for *R*_*a*_ < *R*_0_ < 1 and a third, unstable, equilibrium also exists. Hence, the disease-free equilibrium is only globally stable if 0 < *R*_0_ < *R*_*a*_. (b) Backward bifurcations at other points may also affect the outcome. Although the disease persists for all *R*_0_ > 1 and is eradicated when *R*_0_ < 1 (due to the transcritical bifurcation at *R*_0_ = 1), there is a region *R*_*m*_ < *R*_0_ < *R*_*n*_, where three equilibria coexist. In this region, the outcome depends on the initial conditions.

A backward bifurcation at *R*_0_ = 1 may result in persistence of the disease when *R*_0_ < 1. In this case, the disease will always persist for *R*_0_ > 1. However, there is a point *R*_*a*_ < 1 such that the endemic equilibrium exists for *R*_*a*_ < *R*_0_ < 1 and a third, unstable, equilibrium also exists between the two stable equilibria. Hence, an endemic disease is only eradicated if 0 < *R*_0_ < *R*_*a*_. For *R*_*a*_ < *R*_0_ < 1, the outcome depends on initial conditions. If the disease is still in its early stages—that is, if the initial conditions are sufficiently small—then the system will approach the disease-free equilibrium and the disease will be eradicated. However, if the initial conditions are large, then the system will approach the endemic equilibrium and the disease will persist. Thus, a backward bifurcation prevents the system from switching to the disease-free equilibrium as soon as *R*_0_ < 1 is reached. See Figure 5(a).

Several mechanisms have been shown to lead to backward bifurcation in epidemic models, but backward bifurcations in compartmental models have only recently attracted serious research attention.

Gómez-Acevedo and Li [16] investigated a mathematical model for human T-cell lymphotropic virus type I (HTLV-I) infection of CD4^+^ T cells that incorporates both horizontal transmission through cell-to-cell contact and vertical transmission through mitotic division of infected T cells. They assumed that a fraction *σ* of the infected cells survive the immune system attack after the error-prone viral replication. Under the biologically sound assumptions that the fraction *σ* should be very low and the rate of the mitotic division should be high, their model has a bifurcation that predicts persistent infection for an extended range of the basic reproduction *R*_0_ > *R*_0_(*σ*_0_), where *R*_0_(*σ*_0_) < 1. This model undergoes a backward bifurcation as *σ* increases: multiple stable equilibria exist for an open set of parameter values, when the basic reproduction number is below one.

Safan et al. [17] studied an epidemiological model under the assumption that the susceptibility after a primary infection is *r* times the susceptibility before a primary infection. They present a method for determining the control effort required to eliminate an infection from a host population when subcritical persistence may occur. This effort can be interpreted as a reproduction number but is not necessarily the basic reproduction number.

For *r* > 1 + *μ*/*α*, this model exhibits backward bifurcations, where *μ* is the death rate and *α* is the recovery rate. For such models, the authors presented a method for determining the minimum effort required to eradicate the infection from the endemic steady state if one concentrates on control measures affecting the transmission rate constant.

Garba et al. [18] presented a deterministic model for the transmission dynamics of a single strain of dengue by realistically adopting a standard incidence formulation and allowing dengue transmission by exposed humans and vectors. The model was extended to include an imperfect vaccine for dengue. A backward bifurcation was observed in both models. This makes *R*_0_ < 1 no longer sufficient for effectively controlling dengue in a community. However, this phenomenon can be removed by replacing the standard incidence function in the model with a mass-action formulation.

Reluga et al. [19] proposed a series of epidemic models for waning immunity that can be applied in many different settings. With biologically realistic hypotheses, they found that immunity alone never creates a backward bifurcation. However, this does not rule out the possibility of multiple stable equilibria, which can be shown by a counterexample of the forward bifurcation at *R*_0_ = 1. See Figure 5(b).

## 5. *R*_0_ in Spatial Contexts

When *R*_0_ is considered in a spatial context, many of its properties fail to hold. In particular, diseases with *R*_0_ > 1 can fail to persist, depending on the nature of the spatial transmission. Since many diseases are spatially dependent, this further limits the utility of *R*_0_. As *R*_0_ increases beyond 1, the probability of disease invading the initially infected host group increases, but additional criteria are important to determining the probability of the spreading of the disease to other groups.

### 5.1. Networks

Green et al. [20] related deterministic mean-field models to network models, taking into account the manner in which the contact rate and infectiousness change over time. For a node with exactly *m* connections, the expected number of secondary infections at infection age *u* is given by

(26) $$\begin{array}{c} R\left(m,u\right)=m\left(1-e^{-\beta u/k}\right), \end{array}$$

where *β* is the transmissibility and *k* is the mean number of connections per node. This model applies when the rate of infectious contact is independent of *k*.

If there is a constant rate of generation of new cases, then the expected number of secondary infections in an infectious period of length *u* is given implicitly by

(27) $$\begin{array}{c} R\left(m,u\right)=\overset{u}{\underset{0}{\int }}\left(m-R\right)C_{IS}\left(t\right)\psi \left(t\right)dt, \end{array}$$

where *C*_*IS*_(*t*) denotes the contact per susceptible neighbour and *ψ*(*t*) denotes the infectiousness at time *t* after infection.

Kao [21] showed that novel pathogens may evolve towards a lower *R*_0_, even if this results in pathogen extinction. This is because the presence of exploitable heterogeneities, such as high variance in the number of potentially infectious contacts, increases *R*_0_; thus, pathogens that can exploit heterogeneities in the contact structure have an advantage over those that do not. The exploitation of heterogeneities results in a more rapid depletion of the potentially susceptible neighbourhood for an infected host. While the low *R*_0_ strategy is never evolutionarily stable, invading strains with higher *R*_0_ will also converge to the low *R*_0_ strategy if not sufficiently different from the resident strain. This is in contrast to the conventional belief that the emergence of novel pathogens is driven by maximisation of *R*_0_.

In a randomly mixed epidemiological network, *R*_0_ can be approximated by

(28) $$\begin{array}{cc} R_{0} & =\frac{\langle l_{\text{in}}l_{\text{out}}\rangle }{\langle l_{\text{out}}\rangle }, \end{array}$$

where *l*_in_ and *l*_out_ are, respectively, the number of inward and outward “truly infectious” links per node; the angled brackets represent the expected value of the relevant quantity [22].

Meyers [23] showed that in a contact network framework,

(29) $$\begin{array}{c} R_{0}=T\left(\frac{\langle k^{2}\rangle -\langle k\rangle }{\langle k\rangle }\right), \end{array}$$

where *T* is the mean probability of transmission between individuals and 〈*k*〉 and 〈*k* ^2^〉 are the mean degree and mean square degree of the network. Here, *R*_0_ depends explicitly on the structure of the network (i.e., on 〈*k*〉 and 〈*k* ^2^〉). A single pathogen may, therefore, have very different transmission dynamics depending on the population through which it spreads. If two networks have the same mean degree, *k*, then the one with the larger variance in degree, 〈*k* ^2^〉−〈*k*〉^2^, will be more vulnerable to the spread of disease.

In compartment models, infected hosts are assumed to have potentially disease-causing contacts with random individuals from the population according to a Poisson process that yields an average contact rate of *β* per unit time. The mass-action assumption of compartmental models is tantamount to assuming that the underlying contact patterns form a random graph with a Poisson degree distribution. Estimates of *R*_0_ that assume a mass-action model may, therefore, be invalid for populations with non-Poisson contact patterns and, in particular, will underestimate the actual growth rate of the disease in highly heterogeneous networks.

### 5.2. Individual-Level Models

Rahmandad and Sterman [24] noted that if *R*_0_ > 1 in an agent-based model then, due to the stochastic nature of interactions, it is possible that no epidemic occurs or that it ends early if, by chance, the few initially contagious individuals recover before generating new cases.

Schimit and Monteiro [25] showed that in an individual-level model, *R*_0_ cannot be uniquely determined from some features of transient behaviour of the infective group. The value of *R*_0_ can be unambiguously determined from the asymptotical stable stationary concentrations, but this relies on waiting for the system to reach its permanent regime, which is not feasible in practice. The same value of *R*_0_ can be associated to networks with distinct values of clustering coefficients and average shortest path length. This result can affect the evaluation of the effectiveness concerning different strategies employed for controlling a disease. Because distinct values of topological properties can produce the same value of *R*_0_ in a model considering the spatial structure of the contact network, it is difficult to evaluate the effective contribution of each control measure. This is because the correspondences among *R*_0_ and the topological properties of the contact network are not one-to-one.

### 5.3. Metapopulation Models

Cross et al. [26] showed that when *R*_0_ is based on data collected from simulated epidemics mimicking epidemiological contact-tracing data, *R*_0_ can be substantially greater than one and yet not cause a pandemic. In populations with social or spatial structure, a chronic disease is more likely to invade than an acute disease with the same *R*_0_, because it persists longer within each group and allows for more host movement between groups.

Under the settings where the rate of host population turnover was negligible relative to the rate of disease processes of infection and recovery, they showed that *R*_0_ > 1 was insufficient for disease invasion when the product of the average group size and the expected number of between-group movements made by each individual while infectious was less than 1.

Smith? et al. [15] examined a metapopulation model with travel between two regions, with reproductive ratios *R*_0,1_^(0)^ and *R*_0,2_^(0)^ for each region in the absence of travel, and ${\bar{R}}_{0,1}$ and ${\bar{R}}_{0,2}$ when only susceptibles travel, but infectives do not. They showed that if both *R*_0,1_^(0)^ < 1 and *R*_0,2_^(0)^ < 1, then there are conditions on the travel of susceptible such that ${\bar{R}}_{0,1}>1$ and ${\bar{R}}_{0,2}<1$. Thus, a disease which would otherwise be eradicated in both regions could be sustained in one of the regions if there were sufficient travel of the susceptibles (not infectives). Furthermore, if *R*_0,1_^(0)^ < 1 and *R*_0,2_^(0)^ > 1, then there are conditions on the travel of susceptibles such that ${\bar{R}}_{0,1}>1$ and ${\bar{R}}_{0,2}>1$. Thus, if one region sustains the disease on its own, while the other does not, then sufficient travel of susceptibles (not infectives) could sustain the disease in both regions.

### 5.4. Partial Differential Equation Models

Althaus et al. [27] examined an age-dependent partial differential equation model of in-host HIV infection. They showed that

(30) $$\begin{array}{cc} R_{0} & =\frac{1}{\int_{0}^{\infty }e^{-ra}g\left(a\right)da}, \end{array}$$

where the denominator is the Laplace transform of the generation time distribution *g*(*a*) and *r* is the growth rate. They found that estimates for *R*_0_ were generally smaller than those derived from the standard model when the generation time was taken into account.

## 6. Stochastic Effects

When stochastic effects, such as those inevitably found in nature, are included, the threshold at *R*_0_ = 1 may be disturbed. This includes assumptions about the distribution of transition times (assumed to be exponential in most models) as well as variations in individual parameters.

Heffernan and Wahl [28] derived improved estimates of *R*_0_ for situations in which information about the dispersal of transition times is available to the clinical or epidemiological practitioner. Rather than rederiving *R*_0_ for a number of models (SIR, SEIR, etc.), they introduce a “correction factor”, *ϕ*: the ratio of *R*_0_ when the lifetimes are nonexponentially distributed to the value of *R*_0_ that would be calculated assuming exponential lifetimes. They were able to derive limiting values of *ϕ* and used this to gauge the sensitivity of *R*_0_ to dispersion in the underlying distributions.

By combining the movement of hosts, transmission with groups, recovery from infection and the recruitment of new susceptibles, Cross et al. [29] expanded the earlier analysis of Cross et al. [26] to a much more broader set of disease-host relationships, exploring settings where the duration of immunity ranges from transient to lifelong or where the demographic processes occur on comparable (or faster) timescales to disease processes. The focus of this study was to investigate how recruitment of susceptibles affects disease invasion and how population structure can affect the frequency of superspreading events (SSEs). They found that the frequency of SSEs may decrease with the reduced movement and the group sizes due to the limited number of susceptibles available.

The hierarchical nature of disease invasion in host metapopulations is illustrated by the classification tree analysis of the model results. Firstly, the pathogen must effectively transit within a group (*R*_0_ > 1), and then, the pathogen must persist within a group long enough to allow for movement between different groups. Hence, the infectious period, group size and recruitment of new susceptibles are as important as the local transmission rates in predicting the spread of pathogens across a metapopulation. It should be noted that in 35% of simulations when *R*_0_ was greater than one, the disease failed to invade.

Tildesley and Keeling [30] examined whether *R*_0_ was a good predictor of the 2001 UK Foot and Mouth disease. They concluded that *R*_0_ explained just 29.3% of the standard deviation of the epidemic impact. They also noted that *R*_0_ = 1 did not act as a threshold: at the value of *R*_0_ = 1, only 20% of initial seedings generated epidemics; this probability increased to around 50% for the largest reasonable *R*_0_ values. When heterogeneities exist in the population, infection is most likely to become focused within the high-risk individuals who are both more susceptible and more infectious. This highlights the stochastic nature of the disease in its early stages and the dependence of the ensuing epidemic on favourable local conditions in the neighbourhood of the initial infection.

 The probability of extinction, assuming exponentially distributed infectious periods, was

(31) $$\begin{array}{c} p_{\text{ext}}^{\exp\;}=\frac{1}{R_{0}}, \end{array}$$

when an epidemic began with a single infected individual. Thus, if *R*_0_ ≤ 1, then extinction occurs with probability 1, but if *R*_0_ > 1 then extinction occurs with some probability. See Chiang [31, Chapter 4] for more discussion.

## 7. *R*_0_ Failures

In this section, we note a variety of problems with *R*_0_ that are not covered in the previous sections. These include problems with the underlying structure of compartment models, the mismatch between an individual-based parameter and a population-level compartment model, and the failure of *R*_0_ to accurately measure an outbreak of a new disease.

Breban et al. [32] argued that in order to associate an *R*_0_ to a model of ODEs, an individual-level model (ILM) which is compatible to the ODE model must be developed; only then can the *R*_0_ of the ILM be unambiguously calculated. These ILMs are growing (not static) network models, with individuals added to a network of who infected whom based on global or local network rules. Then, *R*_0_ is computed as the limit of the average number of outgoing links of individuals in a node that no longer accepts new links, as time goes to infinity. They showed that a broad range of *R*_0_ values were compatible with a given ODE model.

For example, consider the basic model

(32) $$\begin{array}{cc} \frac{dS}{dt} & =-\beta I,\\ \frac{dI}{dt} & =\beta I-\mu I, \end{array}$$

where *β* is the transmissibility and *μ* is the disease death rate; note that the transmission term is equivalent to the standard term *βS* *I*/(*S* + *I*) when the depletion of susceptibles is negligible so that *S*/(*S* + *I*) ≈ 1.

The expected *R*_0_ value from this model using other methods (such as the next-generation method) is

(33) $$\begin{array}{c} R_{0}=\frac{\beta }{\mu }. \end{array}$$

The corresponding ILM consists of an infection rule, where an individual joining the infectious pool is infected by an infectious individual who is uniformly randomly selected, and a removal rule, where a uniformly randomly selected individual leaves the infectious pool. The flow of newly infected individuals is *βI*(*t*). Thus, the flow per already-infected individual is *β*. Since the removed individuals are randomly sampled from the infectious individuals, the average length of the infectious period equals the time expectation of the infectious period, which is 1/*β* (rather than 1/*μ*, as calculated from the next-generation method). It follows that *R*_0_ = 1, which is independent of the transmission and death rates from the corresponding ODE. Thus, in this case, *R*_0_ does not signal epidemic growth as anticipated from other methods. 

Roberts [3] noted three fundamental properties commonly attributed to *R*_0_: (i) that an endemic infection can persist only if *R*_0_ > 1, (ii) *R*_0_ provides a direct measure of the control effort required to eliminate the infection, and (iii) pathogens evolve to maximise their *R*_0_ value. He demonstrated that all three statements can be false. The first, as we have noted, can fail due to the presence of backward bifurcations. The second can fail when control efforts are applied unevenly across different host types (such as a high-risk and a low-risk group), since *R*_0_ is determined by averaging over all host types and does not directly determine the control effort required to eliminate infection.

The third can fail when two pathogens coexist at a steady state that exists and is stable whenever both single-pathogen steady states exist but are unstable. In this case, the order in which the pathogens are established in the host population matters. The established parasite has a role in determining a modified carrying capacity and the pathogen with the largest basic reproductive ratio does not necessarily exclude the other.

Breban et al. [33] showed that two individual-level models having exactly the same expectations of the corresponding population-level variables may yield different *R*_0_ values. They showed that obtaining *R*_0_ from empirical contact-tracing data collected by epidemiologists and using this *R*_0_ as a threshold parameter for a population-level model could produce misleading estimates of the infectiousness of the pathogen, the severity of an outbreak, and the strength of the medical and/or behavioural interventions necessary for control. Thus, measuring *R*_0_ through contact tracing (as generally occurs during an outbreak investigation) may not be a useful measure for determining the strength of the necessary control interventions.

Many different individual-level processes can generate the same incidence and prevalence patterns. Thus, assigning a meaningful *R*_0_ value to an ODE model without knowledge of the underlying disease transmission network may be impossible. Only an epidemic threshold parameter can be used to design control strategies. Since *R*_0_ fails to possess this threshold quality, its usefulness may be vastly overstated.

Meyers [23] compared the theoretical calculation of *R*_0_ with observed SARS data from China and showed that estimates of *R*_0_ seemed incompatible. The basic reproductive rate has two critical inputs:

1. intrinsic properties of the pathogen that determine the transmission efficiency per contact and the duration of the infectious period,
2. the patterns of contacts between infected and susceptible hosts in the population.

While the first factor may be fairly uniform across outbreaks, the second may depend significantly on context, varying both within and among populations. The problem with the SARS estimates stems from the mass-action assumption of compartmental models; that is, that all susceptible individuals are equally likely to become infected. When this assumption does not hold, the models may yield inaccurate estimates or estimates that do not apply to all populations. *R*_0_ estimates for SARS in the field were based largely on outbreak data from a hospital and a crowded apartment building, with anomalously high rates of close contacts among individuals.

The author suggested that it might be inappropriate to extrapolate estimates for *R*_0_ from specific settings such as these to the population at large. Conversely, since the population at large is also unlikely to satisfy mass-action requirements, it may also be concluded that *R*_0_ is not a meaningful estimate of disease spread.

## 8. Alternatives to *R*_0_

A number of alternatives have been offered over the years, due to recognised problems with *R*_0_. Older examples include the critical community size [5], the group-level reproductive number, R* [34], the type reproduction number [35] and the basic depression ratio [36]. See Heffernan et al. [2] for a summary of these methods. In this section, we review some of the more recent alternatives to *R*_0_.

The actual reproduction number, *R*_*a*_, is defined as a product of the mean duration of infectiousness and the ratio of incidence to prevalence [37–39]. *R*_0_ coincides with *R*_*a*_ when the transmission probability is constant but accounts for the more general situation when the transmission probability varies as a function of infection age (as happens in diseases such as HIV/AIDS).

 The effective reproduction number *R*(*t*) measures the number of secondary cases generated by an infectious case once an epidemic is underway. In the absence of control measures, *R*(*t*) = *R*_0_ *S*(*t*)/*N*, where *S*(*t*)/*N* is the proportion of the population susceptible [40, 41]. The estimation of reproductive numbers is typically an indirect process because some of the parameters on which these numbers depend are difficult or impossible to quantify directly. The effective reproductive number satisfies *R*(*t*) ≤ *R*_0_, with equality only when the entire population is susceptible [40].

The effective reproduction number is of practical interest, since it is time dependent and can account for the degree of cross-immunity from earlier outbreaks. However, since it is based on the basic reproduction number, the effective reproduction number inherits many of the issues from *R*_0_. 

Breban et al. [32] proposed *Q*_0_, the average number of secondary infections over the infectious population. The average number of secondary infections of actively infectious individuals, *Q*_0_(*t*), is computed as the average number of outgoing links of a node in the infected compartment at time *t*. Then,

(34) $$\begin{array}{c} R_{0}\equiv \underset{t\to \infty }{\lim\;}R_{0}\left(t\right),\\ Q_{0}\equiv \underset{t\to \infty }{\lim\;}Q_{0}\left(t\right), \end{array}$$

when the limits exist. Unfortunately, *R*_0_(*t*) is never defined in the paper, limiting the usefulness of this formulation of *R*_0_. However, by analogy with *Q*_0_(*t*), *R*_0_(*t*) is the average number of outgoing links of a removed compartment at time *t* (Romulus Breban, personal communication).

For the SI model (32), under the assumption that every infection is uniquely assigned as a secondary infection for either a removed or an infected individual,

(35) $$\begin{array}{c} N_{i}\left(t\right)=I\left(t\right)Q_{0}\left(t\right)+N_{r}\left(t\right)R_{0}\left(t\right), \end{array}$$

where *N*_*i*_(*t*) = ∫_0_^*t*^ *βI*(*u*)*du* is the cumulative number of infected individuals that occur in the time interval (0, *t*] and *N*_*r*_(*t*) = ∫_0_^*t*^ *μI*(*u*)*du* is the cumulative number of removed individuals.

Their definition of *R*_0_ evaluates the average number of secondary cases over removed individuals as the distribution of secondary cases becomes stationary. This definition does not imply a particular individual-level model; it depends exclusively on the structure of the disease-transmission network [42]. However, *R*_0_ is not measured at the start of an epidemic, which may limit its usefulness during an initial outbreak.

Grassly and Fraser [43] demonstrated that standard epidemiological theory and concepts such as *R*_0_ do not apply when infectious diseases are affected by seasonal changes. They instead define

(36) $$\begin{array}{c} {\bar{R}}_{0}=D\overset{1}{\underset{0}{\int }}\beta \left(t\right)dt, \end{array}$$

where *β*(*t*) is the transmission parameter at time *t* and *D* is the average duration of infection. Thus, ${\bar{R}}_{0}$ can be interpreted as the average number of secondary cases arising from the introduction of a single infective into a wholly susceptible population at a random time of the year.

They noted that the condition ${\bar{R}}_{0}<1$ is not sufficient to prevent an outbreak, since chains of transmission can be established during high-infectious seasons if *Dβ*(*t*) > 1. However, ${\bar{R}}_{0}<1$ is both necessary and sufficient for long-term disease extinction.

Meyers [23] noted that in the case of networks, estimating the average transmissibility *T* may be more valuable than *R*_0_. This means reporting not only the number of new infections per case, but also the total estimated number of contacts during the infectious period of that case. Given the primary role of contact tracing in infectious disease control, these data are often collected. Unlike *R*_0_, *T* can be justifiably extrapolated from one location to another even if the contact patterns are quite disparate.

Instead of *R*_0_, the author offered a number of alternatives to determine whether an outbreak will occur, based on network modelling. These include the probability that an individual will spark an epidemic and the probability that a disease cluster will spark an epidemic.

The probability that an individual with degree *k* will spark an epidemic is equal to the probability that transmission along at least one of the *k* edges emanating from that vertex will lead to an epidemic. For any of its *k* edges, the probability that the disease does not get transmitted along the edge is 1 − *T*. The probability that disease is transmitted to the attached vertex but does not proceed into a full-blown epidemic is *Tu*, where *u* is the probability that a secondary infection does not spark an epidemic. Thus, the probability that an individual will spark an epidemic is 1 − (1 − *T* + *Tu*)^*k*^.

The probability that an outbreak of size *N* sparks an epidemic is

(37) $$\begin{array}{c} 1-\left(\frac{\sum_{k=1}^{\infty }kp_{k}{\left(1-T+Tu\right)}^{k-1}}{\sum_{k=1}^{\infty }kp_{k}}\right), \end{array}$$

where *p*_*k*_ is the relative frequency of vertices of degree *k* in the network.

Kao et al. [22] defined an epidemiological network contact matrix *M* whose elements *m*_*ij*_ are either 1 or 0, depending on whether an infectious contact between nodes *i* and *j* is possible. The spectral radius of *M* is an alternative approximation for *R*_0_, which can be calculated via a weighted version of (28).

This explicitly accounts for the full contact structure of the network, but the evaluation of extremely large, reasonably dense matrices (some highly active nodes may have hundreds of potentially infectious links) is difficult and time consuming, particularly when this evaluation process must be repeated multiple times. However, comparisons between the two approximations for subsets of a sheep network with several thousand nodes show little difference between *R*_0_ and the spectral radius of *M* (typically less than 5%).

Kamgang and Sallet [44] used the special structure of Metzler matrices (real, square matrices with nonnegative off-diagonal entries) to define *𝒯*_0_, an analytical threshold condition. *𝒯*_0_ is a function of the parameters of the system such that if *𝒯*_0_ < 1, the disease-free equilibrium is locally asymptotically stable, and if *𝒯*_0_ > 1, the disease-free equilibrium is unstable. *𝒯*_0_ has an association with *R*_0_, although it is a stronger condition; however, it has no direct biological interpretation. The algorithm for deriving *𝒯*_0_, although highly mathematical in nature, allows computation of a threshold for high-dimensional epidemic models.

Huang [45] defined four reproductive numbers associated with four types of transmission patterns, each depending on *z*, the ratio of the mean infectious period to the mean latent period. These four reproduction numbers are the following:

1. *R*_0_^*I*^, the minimal reproductive number associated with the slowest latency process and the fastest recovery process,
2. *R*_0_^*II*^, the middle reproductive number associated with mean latency and recovery processes,
3. *R*_0_^*III*^, the maximal reproductive number associated with the fastest latency process and the slow recovery process,
4. *R*_0_^*IV*^, the largest reproductive number associated with the fastest latency process and the extremely slow recovery process.

All four reproduction numbers are strictly increasing functions of *z* and satisfy

(38) $$\begin{array}{c} R_{0}^{I}<R_{0}^{II}<R_{0}^{III}<R_{0}^{IV}. \end{array}$$

These numbers allow a disease to be classified as mild (*R*_0_^*I*^ < *R*_0_ < *R*_0_^*II*^) or severe (*R*_0_^*III*^ < *R*_0_ < *R*_0_^*IV*^).

Hosack et al. [46] noted that *R*_0_ does not necessarily address the dynamics of epidemics in a model that has an endemic equilibrium. They used the concept of reactivity to derive a threshold index for epidemicity, *E*_0_, which gives the maximum number of new infections produced by an infective individual at a disease-free equilibrium. They also showed that the relative influence of parameters on *E*_0_ and *R*_0_ may differ and lead to different strategies for control.

If *R*_0_ is derived from the next-generation operator so that

(39) $$\begin{array}{cc} R_{0} & =\rho \left(FV^{-1}\right), \end{array}$$

then the threshold for endemicity is

(40) $$\begin{array}{cc} E_{0} & =\rho \left(\frac{F+F^{T}}{2}V^{-1}\right), \end{array}$$

such that the system is reactive when *E*_0_ > 1 and nonreactive when *E*_0_ < 1. *E*_0_ describes the transitory behaviour of disease following a temporary perturbation in prevalence. If the threshold for epidemicity is surpassed, then disease prevalence can increase further even when the disease is not endemic. This suggests that epidemics can occur even in areas where long-term transmission cannot be maintained.

Reluga et al. [47] defined the discounted reproductive number *R*_*d*_. The discounted reproductive number is a measure of reproductive success that is an individual's expected lifetime offspring production discounted by the background population growth rate. It is calculated as

(41) $$\begin{array}{cc} R_{d} & =\rho \left(F{\left(\delta I-V\right)}^{-1}\right), \end{array}$$

where *δ* is the discount rate, *I* is the identity matrix, and *F* and *V* are from the next-generation matrix decomposition. *R*_*d*_ combines properties of both the basic reproductive number and the ultimate proliferation rate although it also inherits the nonuniqueness problems from the next-generation method.

Nishiura [4] developed a likelihood-based method for estimating *R*_0_ without assuming exponential growth of cases and offers a corrected value of the actual reproduction number. The author noted that *R*_0_ is extremely sensitive to dispersal of the progression of a disease or variations in the underlying epidemiological assumptions.

## 9. Discussion

Despite being a crucial concept in disease modelling, with a long history and frequent application, *R*_0_ is deeply flawed. It is not a measure of the number of secondary infections, it is never calculated consistently, and it fails to satisfy the threshold property. Rarely has an idea so erroneous enjoyed such popular appeal. Why, then, are we so attached to the concept?

The answer is that *R*_0_, despite all its flaws, is all that we have. No other concept has so effectively transcended mathematics, biology, epidemiology, and immunology. No other concept is so general that it can be understood in terms of compartment models, network models, partial differential equations, and metapopulation models. “The number of secondary infections” has an intuitive appeal that outlasts even the inaccuracy of that statement when applied to the concept.

The threshold nature of *R*_0_ is used to monitor and control severe real-time epidemics; control measures are often concluded if *R*_0_ < 1 [48], making the problems with *R*_0_ more than just theoretical. Due to the inconsistencies in calculation, different diseases cannot be compared unless the same method was used to calculate *R*_0_; if HIV has an *R*_0_ of 3 and swine flu has an *R*_0_ of 4, we cannot conclude that swine flu is worse than HIV if different methods were used to determine these values. All we can conclude is that both diseases have *R*_0_ > 1; however, as we have demonstrated, this does not necessarily guarantee disease persistence.

Of the many different methods used to calculate *R*_0_, only the survival function reliably calculates the average number of secondary infections; however, this method is too cumbersome to use in most practical settings. The next-generation method is probably the most popular, yet it suffers from uniqueness problems and does not cope well with more than one disease state. Since *R*_0_ is rarely measured in the field, it instead relies upon after-the-fact calculations to determine the strength of disease spread [2]. This limits its usefulness even further.

Policy decisions are being based upon the concept, with limited understanding of the complexity and errors that exist in the very structure of the concept. Funding decisions about where money should be best spent are based on estimates of *R*_0_, resources are directed towards one disease over another, and monitoring programmes are abandoned, their objectives only half-realised, because of *R*_0_. Lives may be saved or lost, based on this imperfect and inconsistent measure.

*R*_0_ is a quantity that relates to the initial phase of an epidemic. This makes practical sense in terms of disease prevention. However, it is also used to guide eradication efforts when a disease is endemic. Some methods derive an eradication threshold from an equilibrium value that may not be attained for a very long time. This suggests that different measures are needed in different stages of an epidemic in order to characterise transmissibility and to guide intervention strategies. When a new pathogen emerges, a quantity describing the initial spread is useful. When a disease is endemic, a quantity that applies to the long-term equilibrium is more appropriate.

We note that although the definition of *R*_0_ is broad, it is not a universal quantity that applies to all settings. In different settings, one should use a quantity that satisfies the following properties: that an endemic equilibrium only persist if *R*_0_ > 1 and that *R*_0_ provides a direct measure of the control effort required for eradication. The contact structure of the population, the variation of risk factors and the order of establishing parasites (if applicable) should accompany the identification of a meaningful *R*_0_. 

What is urgently needed is a simple, but accurate, measure of disease spread that has a consistent threshold property and which can be understood by nonmathematicians. If *R*_0_ is to be used, it must be accompanied by a declaration of which method was used, which assumptions are underlying the model (e.g. mass-action transmission) and evidence that it is actually a threshold, with no backward bifurcation. Without such caveats, the concept of *R*_0_ will continue to fail.

## Appendix

### A. *R*_0_ for Specific Diseases

In this appendix, we illustrate some recent calculations of *R*_0_ for various diseases from the past few years, noting in particular specific values for *R*_0_ that have been given. This illustrates the wide variety of values that are presented as being “the” *R*_0_ value for a specific disease.

#### A.1. HIV

Bacaër et al. [49] developed a mathematical model of the HIV/AIDS epidemic in Kunming, China. They considered needle sharing between injecting drug users and commercial sex between female sex workers and clients. The basic reproductive ratio is expressed as

(A.1) $$\begin{array}{cc} R_{0} & \approx \max\;\left\{R_{0}^{\text{IDU}},R_{0}^{\text{sex}}\right\}, \end{array}$$

where the superscript “IDU” represents injecting drug users and “sex” represents sex workers. They estimate *R*_0_^IDU^ = 32 and *R*_0_^sex^ = 1.7.

Abu-Raddad et al. [50] examined the population-level implications of imperfect HIV vaccines. They divided the population into two groups: unvaccinated and vaccinated, both of whom could become infected (albeit at different rates, depending on the efficacy of the vaccine). By forming two independent next-generation matrices, they derived *R*_0_ and *R*_0*V*_, the basic reproductive ratios for the unvaccinated and vaccinated populations, respectively. They note that the condition *R*_0*V*_ < 1 may not be sufficient for disease eradication, due to the possibility of a backward bifurcation. The two formulas are equivalent in the absence of the vaccine, assuming that risky behaviour does not change. They estimate *R*_0*V*_ = 1.4 in the absence of vaccine protection.

Gran et al. [39] argued that *R*_0_ was inappropriate for long-lasting infections and instead defined the actual reproduction number *R*_*a*_(*t*). This is a time-dependent measure defined as the average number of secondary cases per case to which the infection was actually transmitted during the infectious period in a population. Thus,

(A.2) $$\begin{array}{c} R_{a}\left(t\right)=\frac{X\left(t\right)}{Y\left(t\right)}D, \end{array}$$

where *X*(*t*) is the incidence rate at time *t*, *Y*(*t*) is the prevalence at time *t*, and *D* is the average length of the infectious period. They illustrated their results for a staged HIV/AIDS progression model for homosexual men in the UK and showed that *R*_*a*_ was 13.81 and 17.01 in 1982 and 1983, respectively, but was much closer to 1 by the mid 1990s, due to decreases in risk behaviour.

Smith? et al. [15] used the nonuniqueness of *R*_0_-like thresholds to evaluate the feasibility of eradicating AIDS using available resources. They developed a linear metapopulation model that has the same eradication threshold as more realistic models. They defined the threshold as

(A.3) $$\begin{array}{c} T_{0}=e^{s(K)}, \end{array}$$

where *s*(*K*) is the maximal real part of all eigenvalues of *K*, the Jacobian matrix evaluated at the disease-free equilibrium; *K* represents the change of status of infected individuals in different regions. The model has the property that the disease will be eradicated if *T*_0_ < 1 but will persist if *T*_0_ > 1. The paper stresses that this quantity should be understood only as a threshold condition for eradication; the model from which it derives does not quantify the transient dynamics, the timecourse of the infection or the prevalence of the disease. However, they showed that the simpler version of the model has the same eradication threshold (*T*_0_) as more realistic, nonlinear models. The mathematical analysis results, together with a simple cost analysis, used the threshold nature of *R*_0_ to show that eradication of AIDS is feasible, using the tools that we have currently to hand, but action needs to occur immediately and globally.

#### A.2. Influenza

Chowell et al. [51] used four methods to calculate *R*_0_ using data from the 1918 influenza pandemic in California. The four methods involved (1) an early exponential growth rate, (2) an SEIR model, (3) a more complicated SEIR model with asymptomatic and hospitalised cases, and (4) a stochastic SIR model with Bayesian estimation. These yielded average *R*_0_ values of (1) 2.98, (2) 2.38, (3) 2.2, and (4) 2.1 during the first 17 days of the epidemic and then 2.36 for the entire autumn wave. In particular, uncertainty during the early part of the epidemic leads to a wide range of estimates for *R*_0_ (0.5–3.5, 95% confidence interval), but uncertainty decreases with more observations.

Wallinga and Lipsitch [52] investigated how generation intervals shape the relationship between growth rates and reproductive numbers. For new emerging infectious diseases, the observed exponential epidemic growth rate *r* can indirectly infer the value of the reproductive number. The existence of a few different equations that relate the reproductive number to the growth rate makes such inference ambiguous. It is unclear which of these equations might apply to new infection. The authors showed that these different equations differ only with respect to their assumed shape of the generation-interval distribution. They found that the shape of the generation-interval distribution determines which equation is appropriate for inferring the reproductive number from the observed growth rate. By assuming all generation intervals to be equal to the mean, they obtained an upper bound on the range of possible values that the reproductive number may attain for a given growth rate. They also showed that it is possible to obtain an empirical estimate of the reproductive number by taking the generation-interval distribution equal to the observed distribution.

The authors illustrated the impact that various assumptions about the shape of the generation interval distribution may have on the estimated value of the reproduction numbers for a given growth rate using human infections with influenza A virus. Observed generation intervals for influenza A in a Japanese household study excluding possible coprimary and tertiary cases [53] estimated a mean generation interval of 2.85 days and a standard deviation of 0.93 days. Without specific assumptions about the shape of the generation-interval distribution, they found that the reproductive number of influenza A is larger than *R* = 1, but smaller than *R* = 1.77.

Chowell and Nishiura [54] reviewed quantitative methods to estimate the basic reproductive ratio of pandemic influenza. They use the intrinsic growth rate to estimate *R*_0_ in the range from 1.36 to 2.07. They defined the effective reproduction rate

(A.4) $$\begin{array}{cc} R\left(t\right) & =\overset{\infty }{\underset{0}{\int }}A\left(t,\tau \right)d\tau , \end{array}$$

where *A*(*t*, *τ*) is the reproductive power at time *t* and infection age *τ* at which an infected individual generates secondary cases. If contact and recovery rates do not vary with time, then this simplifies to

(A.5) $$\begin{array}{c} R\left(t\right)=\frac{S\left(t\right)}{S\left(0\right)}R_{0}, \end{array}$$

where *S*(*t*) is the population of susceptibles at time *t*.

They also outlined statistical methods of estimation and showed that the expected number of secondary transmissions is given by

(A.6) $$\begin{array}{cc} R_{0} & =\overset{\infty }{\underset{k=1}{\sum }}kp_{k}, \end{array}$$

where the pattern of secondary transmission follows a discrete probability distribution *p*_*k*_ with *k* secondary transmissions. This approach accounts for demographic stochasticity, which can be crucial during the initial phase of an epidemic when the number of infected individuals is small. This has been applied to observed outbreak data for avian influenza, where extinction was observed before growing to a major epidemic [55].

Tiensin et al. [56] used flock-level mortality data and statistical back-calculation methods to estimate *R*_0_ from an SIR model for the 2004 avian influenza epidemic in Thailand. They calculated *R*_0_ to be between 2.26 and 2.64, depending on the length of the infectious period.

#### A.3. Malaria

Smith et al. [57] revisited the basic reproduction number of malaria and its implications for malaria control. Their estimates of *R*_0_ are based on two more commonly measured indices called the entomological inoculation rate, which is the average number of infectious bites received by a person in a year, and the parasite ratio, which is the prevalence of malaria infection in humans. They made 121 estimates of *R*_0_ for * Plasmodium falciparum* malaria in African populations. The estimates (which do not come from a mathematical model) range from around one to more than 3000; the median is 115 and the interquartile range is 30–815.

They then revised the formula of *R*_0_ to adopt potential sources of bias when biting is heterogeneous and when there is some transmission-blocking immunity. Under the assumption that the transmission-blocking immunity develops, the estimates of *R*_0_ ranged from below one to nearly 11,000, with a median of 86 and an interquartile range of 15–1,000. This is because transmission-blocking immunity and heterogeneous biting skew the probability that a mosquito becomes infected, per bite. They also showed that in small human populations, the classical formulas of *R*_0_ approximate transmission when counting infections from mosquito to mosquito after one parasite generation, but they overestimate it from human to human.

#### A.4. West Nile Virus

Wonham et al. [11] examined the conflicting outbreak predictions for West Nile virus, showing how the choice of transmission term affects *R*_0_. They concluded that some transmission terms apply biologically only at certain population densities and showed that six common North American bird species would be effective outbreak hosts. All seven models considered shared a similar compartment structure, but with varying factors, such as incubation periods, loss of immunity, age structure, and so forth. They created a core model synthesised from all seven models and used the next-generation method to calculate

(A.7) $$\begin{array}{c} R_{0}=\sqrt{\overset{\underset{}{\underset{︸}{\frac{\beta_{R}}{d_{V}}}}}{\text{vector to reservoir}}\times \overset{\underset{}{\underset{︸}{\frac{\beta_{R}N_{V}^{*}}{d_{R}N_{R}^{*}}}}}{\text{reservoir to vector}}}, \end{array}$$

where *β*_*R*_ is the transmissibility of the reservoir, *d*_*R*_ is the death rate of the reservoir, *d*_*V*_ is the death rate of the vector, *N*_*V*_* is the total vector population at the disease-free equilibrium and *N*_*R*_* is the total reservoir population at the disease-free equilibrium.

The first term under the square root sign represents the number of secondary reservoir infections caused by one infected vector. The second term under the square root sign represents the number of secondary vector infections caused by one infected reservoir. The square root gives the geometric mean of the two terms (see Section 2). Using reported minimum, mean and maximum estimates, 10,000 Monte Carlo estimates were run to generate an estimated distribution of *R*_0_. Mean numerical values for *R*_0_ for house sparrows ranged from 25–1200.

However, these values are enormous, implying that a single sparrow would infect up to 1.44 million sparrows (i.e., 1200 × 1200) during its infectious lifetime. Cruz-Pacheco et al. [58], for example, estimated *R*_0_ = 5.08 for the house sparrow, using data from Komar et al. [59].

#### A.5. Cholera

Hartley et al. [60] developed a compartment model of cholera incorporating both a hyperinfective state and a lower infective state. They used the average age of infection to estimate *R*_0_ from data for four cholera outbreaks in developing countries during the nineties. *R*_0_ values ranged from 3.1 in Indonesia (1993–1999) to 15.3 in Pakistan (1990–1995), with an average of 8.7. They then used the next-generation method to develop a theoretical estimate for *R*_0_ that incorporates hyperinfectivity. They show that this raises *R*_0_ from approximately 3.2 when there is no hyperinfectivity to 18.2 when the contact with hyperinfected water is similar to contact with nonhyperinfected water, but that *R*_0_ could be significantly larger with more frequent contact with hyperinfected water.

#### A.6. Dengue

Nishiura [61] clarified the contributions of mathematical and statistical approaches to dengue epidemiology. This highlighted the practical importance of the basic reproduction number, *R*_0_, in relation to the critical proportion of vaccination required to eradicate the disease in the future. The author illustrated three different methods to estimate *R*_0_: (i) the final size equation, (ii) the intrinsic growth rate, and (iii) age distribution, with published estimates and examples. The author pointed out that, although the estimates of *R*_0_ most likely depend on the ecological characteristics of the vector population, it would be appropriate to assume a serotype-nonspecific estimate for *R*_0_ is approximately 10, at least when planning vaccination strategies in endemic areas.

#### A.7. Rift Valley Fever

Gaff et al. [12] modelled Rift Valley fever, a mosquito-borne disease affecting both humans and livestock that currently exists in developing countries but has the potential to be introduced to the western world. The disease can be transmitted both horizontally (from host species to host species via mosquitoes) or vertically (via parent-to-offspring transmission in mosquitoes). They expressed the basic reproductive ratio as

(A.8) $$\begin{array}{c} R_{0}=R_{0,V}+R_{0,H}, \end{array}$$

where *R*_0,*V*_ is the basic reproductive ratio for vertical transmission and *R*_0,*H*_ is the basic reproductive ratio for horizontal transmission. They used the next-generation method and uncertainty analysis to calculate *R*_0_ = 1.19, with a range from 0.037 to 3.743.

#### A.8. Bluetongue

Gubbins et al. [13] examined bluetongue virus, an insect-borne infectious disease of ruminants in Europe. They used the next-generation method to determine *R*_0_ and then Latin Hypercube sampling and partial rank correlation coefficients to assess the effect of parameter variation. Median values for *R*_0_ were 0.81 (cattle only), 0.73 (sheep only) and 0.55 (both cattle and sheep), implying that the disease should not persist. However, the corresponding maximum values were 18.77, 15.48, and 12.82, respectively, suggesting that variation in parameters can have an enormous impact on the outcome, including persistence of the disease. The most significant changes in the estimation of *R*_0_ were due to the mosquito biting rate, the extrinsic vector incubation period, the vector mortality rate, the probability of transmission from host to vector, and the ratio of vectors to cattle and sheep.

#### A.9. Plant Pathogens

van den Bosch et al. [6] described a systematic method to calculate the basic reproduction ratio from knowledge of a pathogen's life cycle and its interactions with the host plant. They developed a system of linear difference equations and rearranged the dominant eigenvalue to find *R*_0_. A fraction *q* of infected spores are deposited in location 1, while a fraction 1 − *q* are deposited in location 2. This results in

(A.9) $$\begin{array}{c} R_{0}=q\left(\gamma_{1}\alpha_{1}\tau_{1}\rho H\right)+\left(1-q\right)\left(\gamma_{2}\alpha_{2}\tau_{2}\rho H\right), \end{array}$$

where the first term represents the number of spores that successfully germinate in location 1 and the second term represents the number of spores that successfully germinate in location 2. Here, *γ*_*i*_ is the probability that a spore deposited on location *i* will germinate, *α*_*i*_ is the number of spores produced per time unit on location *i*, *τ*_*i*_ is the infectious period of a spore-producing lesion on location *i*, *ρ* is the probability that a spore is deposited on a susceptible site, and *H* is the density of susceptible sites in a host population.

They included a separate box explaining pitfalls in calculating the basic reproductive ratio from nonlinear models and noted its nonuniqueness, calculating two distinct values for *R*_0_. However, they were unable to decide which value was correct. The authors conclude by stating that “Although nonlinear differential equations are invaluable tools for studying epidemics, they should not serve as the primary basis for the determination of *R*_0_.”

Cunniffe and Gilligan [62] considered the effects of host demography upon the outcomes for invasion, persistence and control of pathogens in a model for botanical epidemics. They used the next-generation method to calculate

(A.10) $$\begin{array}{c} R_{0}=R_{0}^{p}+R_{0}^{s}, \end{array}$$

partitioned into primary and secondary infection. These values are unaffected by host demography. However, the endemic level of infection is highly sensitive to changes in *R*_0_ when *R*_0_ > 1. If *R*_0_ is increased by shortening the infectious period, then the endemic level of infection increases monotonically. However, if increases in transmission rates or decreases in the decay of free-living inoculum increase *R*_0_, then the endemic level of infection first increases (for 1 < *R*_0_ < 2) but then decreases (for *R*_0_ > 2). Consequently, increasing the intensity of control can result in more endemic infection.
